# Hierarchical machine learning model predicts antimicrobial peptide activity against *Staphylococcus aureus*

**DOI:** 10.3389/fmolb.2023.1238509

**Published:** 2023-09-18

**Authors:** Hosein Khabaz, Mehdi Rahimi-Nasrabadi, Amir Homayoun Keihan

**Affiliations:** ^1^ Molecular Biology Research Center, Systems Biology and Poisonings Institute, Baqiyatallah University of Medical Sciences, Tehran, Iran; ^2^ Faculty of Pharmacy, Baqiyatallah University of Medical Sciences, Tehran, Iran

**Keywords:** *Staphylococcus aureus*, antimicrobial peptides, machine learning, antimicrobial activity, classification model

## Abstract

**Introduction:** *Staphylococcus aureus* is a dangerous pathogen which causes a vast selection of infections. Antimicrobial peptides have been demonstrated as a new hope for developing antibiotic agents against multi-drug-resistant bacteria such as *S. aureus*. Yet, most studies on developing classification tools for antimicrobial peptide activities do not focus on any specific species, and therefore, their applications are limited.

**Methods:** Here, by using an up-to-date dataset, we have developed a hierarchical machine learning model for classifying peptides with antimicrobial activity against *S. aureus*. The first-level model classifies peptides into AMPs and non-AMPs. The second-level model classifies AMPs into those active against *S. aureus* and those not active against this species.

**Results:** Results from both classifiers demonstrate the effectiveness of the hierarchical approach. A comprehensive set of physicochemical and linguistic-based features has been used, and after feature selection steps, only some physicochemical properties were selected. The final model showed the F1-score of 0.80, recall of 0.86, balanced accuracy of 0.80, and specificity of 0.73 on the test set.

**Discussion:** The susceptibility to a single AMP is highly varied among different target species. Therefore, it cannot be concluded that AMP candidates suggested by AMP/non-AMP classifiers are able to show suitable activity against a specific species. Here, we addressed this issue by creating a hierarchical machine learning model which can be used in practical applications for extracting potential antimicrobial peptides against *S. aureus* from peptide libraries.

## 1 Introduction

*Staphylococcus aureus* is a prominent human pathogen that causes a wide range of infections, including pleuropulmonary, osteoarticular, skin, and soft tissue infections. In the United States, nearly 50 percent of deaths caused by antibiotic-resistant bacterial pathogens are attributed to *methicillin-resistant S. aureus* (MRSA) infections (Stryjewski and Chambers, 2008; Mohamed et al., 2016). It has been reported that *S. aureus* was the leading bacterial cause of death in 135 countries and had the highest mortality rate in individuals aged above 15 years worldwide (Ikuta et al., 2022). The evasion of staphylococcal biofilms and toxins can result in prolonged inflammation, chronic infections, and delayed wound healing (Wolcott et al., 2010).

Antimicrobial peptides (AMPs) are a group of naturally occurring or synthetic short peptides with the ability to kill bacterial cells. AMPs typically interact with bacterial membranes, making it difficult for bacteria to develop resistance against them (Zasloff, 2002; Hancock and Sahl, 2006). This has generated increased interest in AMPs as potential substitutes for conventional antibiotics as antibiotic resistance has become a global crisis (Hancock and Sahl, 2006; Chen and Lu, 2020).

Numerous experimental reports have identified AMPs with antimicrobial activity against *S. aureus*, which are accessible in online databases. However, experimental studies on peptides are often costly and time-consuming (Lee et al., 2017). In contrast, computational approaches, particularly machine learning techniques, have enabled the development of high-throughput models for predicting various aspects of AMP functionality, including antimicrobial activity (Vishnepolsky and Pirtskhalava, 2014; Lee et al., 2017) and toxicity against human cells (Chaudhary et al., 2016; Kleandrova et al., 2016).

Most studies in the field of AMP classification have primarily focused on distinguishing AMPs from non-AMPs (Vishnepolsky and Pirtskhalava, 2014; Kleandrova et al., 2016). However, due to the significant diversity among target bacteria, the functionality of AMPs could vary greatly among different bacterial families. Therefore, simply predicting a peptide as an AMP does not necessarily imply that it will exhibit activity against a specific bacterial family. This limitation hinders the applicability of general studies in real-world challenges. Vishnepolsky et al. (2018) developed predictive models to classify AMPs active against Gram-negative bacteria, specifically *Escherichia coli*. Speck-Planche et al. introduced a multi-target model to identify AMPs active against Gram-positive pathogens, including *S. aureus* (Speck-Planche et al., 2016). However, both studies predominantly utilized datasets composed of strong and weak AMPs. Since AMPs represent a small fraction of the peptide space, models trained solely on this limited portion have limited applicability to peptides that deviate significantly from AMP characteristics.

A hierarchical machine learning model refers to a classification approach that involves a multi-level process of classification, where the output of one classifier serves as the input for another (Wehrmann et al., 2018). Hierarchical classifiers have been previously used for several function prediction models such as gene function prediction (Valentini, 2010) and protein function prediction (Eisner et al., 2005). Here, we use machine learning approaches to construct a hierarchical model that discriminates AMPs with specific antimicrobial activity against *S. aureus* by incorporating physicochemical and linguistic-based properties of peptides.

## 2 Materials and methods

### 2.1 Preparing data

Two datasets were needed for this study. Dataset-1 includes AMP (positive) and non-AMP (negative) sets. The positive set was created using AMP records from the Database of Antimicrobial Activity and Structure of Peptides (DBAASP) (Gogoladze et al., 2014). Records with D-amino acids, unnatural residues, C-terminal modifications (except the amid group), and N-terminal modifications (except for acetyl) were removed from the dataset. Moreover, peptide sequences shorter than six residues and longer than 50 residues were also removed since there was no sufficient data in those ranges. All concentrations reported with the μg/mL unit were converted to μM using peptide molecular weight. Only records with a reported minimum inhibitory concentration (MIC) =< 15 μM were used in the final dataset. As for the negative set, the dataset created by Gabere and Noble (2017) was used with the same restrictions. Highly similar sequences (maximum 90%) were removed using CD-HIT software (Huang et al., 2010). The final dataset included 2,144 AMPs and 2,144 non-AMPs. The dataset was split into three sets of train (64%), validation (16%), and test (20%) with no overlapping records and enabled stratify argument on the class label.

Dataset 2 included AMP records with reported activity against *S. aureus* from DBAASP. Peptides with an MIC of 10 µM or lower were labeled as positive, and peptides with an MIC of 15 µM or higher were labeled as negative. Most AMPs have more than one reported activity in the database. After labeling every record, peptides for which all their records had the same label were kept in the dataset. In other words, if a peptide had mixed positive and negative labels in its activity records, it would be excluded from the dataset. The final dataset 2 included 2,488 positive and 1,595 negative AMPs. Train, validation, and test sets were constructed as mentioned previously.

### 2.2 Feature extraction

For dataset 1, a total of seven physicochemical properties, namely, hydrophobicity, net charge, molecular weight, charge density, isoelectric point, hydrophobic moment, and aggregation propensity *in vivo*, were extracted. For dataset 2, a more complex set of features was extracted. A total of 1,527 physicochemical and linguistic-based properties, including autocorrelation, physiochemical composition, transition, and distribution, were extracted from AMP sequences using Propy Python library (Cao et al., 2013) (Supplementary Table S1). All peptide records from DBAASP have four properties of net charge, normalized hydrophobic moment, normalized hydrophobicity, and isoelectric point. Charge densities were calculated similar to the previous work (Vishnepolsky and Pirtskhalava, 2014) using net charge and molecular weight.

### 2.3 Feature selection strategy

Feature selection was carried out in dataset 2 due to its high number of features. First, using Mathematica software (Wolfram Research, 2020), the Pearson correlation coefficient between all pairs of features was calculated, and then, features with a correlation over 95% were grouped together. From each set of the correlated group, only one feature was kept. To select relevant features, the SelectFromModel meta-transformer of scikit-learn library (Pedregosa et al., 2011) was used. The training set was split into five parts with no overlapping records. Then, random forest classifiers were trained on each part independently, and important features were extracted from each classifier. Features shared among all five parts were considered important and kept for further usage.

### 2.4 Training classification models

Classification models were trained in datasets 1 and 2 independently. The test step was also carried out separately. Several learning algorithms, including random forest, support vector classification (SVC), linear SVC, K-nearest neighbors, and naïve Bayes, were trained using 10-fold cross validation and grid search to optimize hyper-parameters for each algorithm. All algorithms were optimized to obtain the highest F1-scores and the best performance on the validation set. Multiple combinations of hybrid-voting classifiers were also constructed using the best obtained models in dataset 2. The performance of the final models was evaluated on the test set. The comparison of performances was carried out using performance measures including precision, recall, F1-score, accuracy, AUC, and hamming distance.

To evaluate the performance of the final package, a new dataset including AMPs and non-AMPs was used as an independent test. New AMP activity data against *S. aureus* were obtained from DBAASP and processed as mentioned in Methods (such as natural residues, specific sequence length, and no terminal modifications). All AMPs were introduced in 2022–2023 and were new for the model. Finally, a total of 118 new peptides including 59 non-AMPs and 59 AMPs with reported activity against *S. aureus* were tested. For final performance measurements, non-AMPs and AMPs not active against *S. aureus* were considered “negative,” and AMPs active against *S. aureus* were considered “positive.”

## 3 Results

### 3.1 Susceptibility comparison

The susceptibility of *S. aureus*, *P. aeruginosa*, and *E. coli* to 1,398 AMPs was investigated using the MIC values reported in the DBAASP dataset. Results are shown in Table 1. It can be seen that more than 28% of AMPs have more than 20 µM difference in the MIC against *S. aureus* and *P. aeruginosa*. Even *E. coli* and *P. aeruginosa*, which are both Gram-negative, show very different results. If we use these MIC values to label these AMPs with high antimicrobial activity and low antimicrobial activity labels, the results are going to be highly varied for different species. More than 32% of AMPs obtained opposite labels for *P. aeruginosa* and *S. aureus*.

**TABLE 1:** Comparison of susceptibility of *S. aureus*, *P. aeruginosa*, and *E. coli* to similar AMPs.

Comparison	Fraction of AMPs with more than 10 µM difference in MIC	Fraction of AMPs with more than 20 µM difference in MIC	Fraction of AMPs with more than 50 µM difference in MIC	Hamming distance	Percentage of AMPs with opposite activity label (%)
*S. aureus* vs	0.4134	0.2847	0.118	452	32.33
*P. aeruginosa*					
*S. aureus* vs	0.3376	0.2310	0.0937	384	27.47
*E. coli*					
*P. aeruginosa* vs	0.3541	0.2389	0.0973	380	27.18
*E. coli*					

### 3.2 Performance of the AMP/non-AMP classification

**FIGURE 1:**
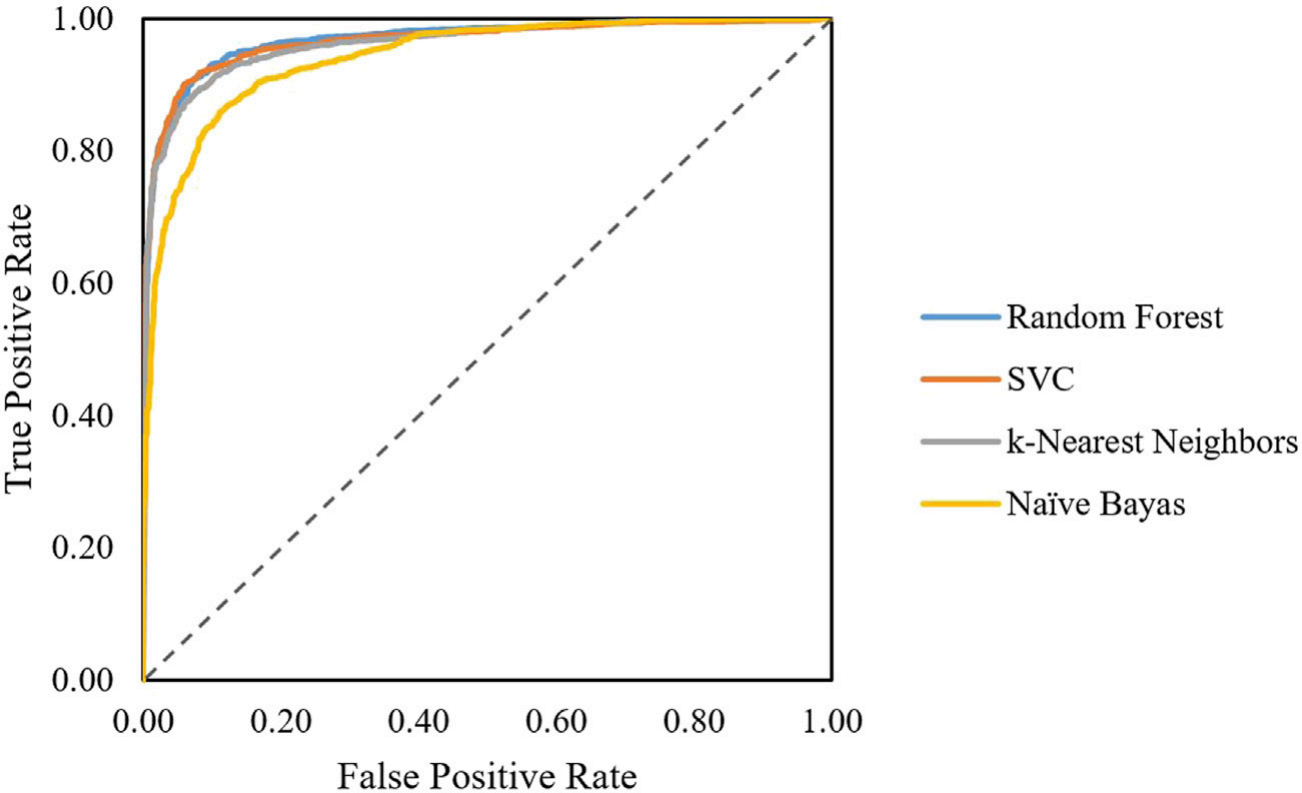
ROC curves obtained for each algorithm classifying AMPs from non-AMPs.

The first classification model in the pipeline was trained using random forest, SVC, linear SVC, K-nearest neighbors, and naïve Bayes algorithm. Performance on the train set can be evaluated by ROC curves (Figure 1). The dotted line shows the performance of a completely random classifier. The larger area under curve in the ROC curve corresponds to a better performance. Models with random forest, SVC, and KNN show very good performances compared to naïve Bayes. The performance of models on test sets was evaluated (Table 2). The results show that SVC, random forest, and KNN with accuracy and the F1-score above 0.9 show great performances for classifying AMPs and non-AMPs.

**TABLE 2:** Performance of AMP/non-AMP classifiers with different algorithms on the test set.

Algorithm	Precision	Recall	F1-score	Accuracy
Random forest	0.919	0.917	0.918	0.918
SVC	0.945	0.903	0.923	0.925
KNN	0.925	0.901	0.913	0.914
Naïve Bayes	0.845	0.886	0.865	0.862

### 3.3 Feature selection

**FIGURE 2:**
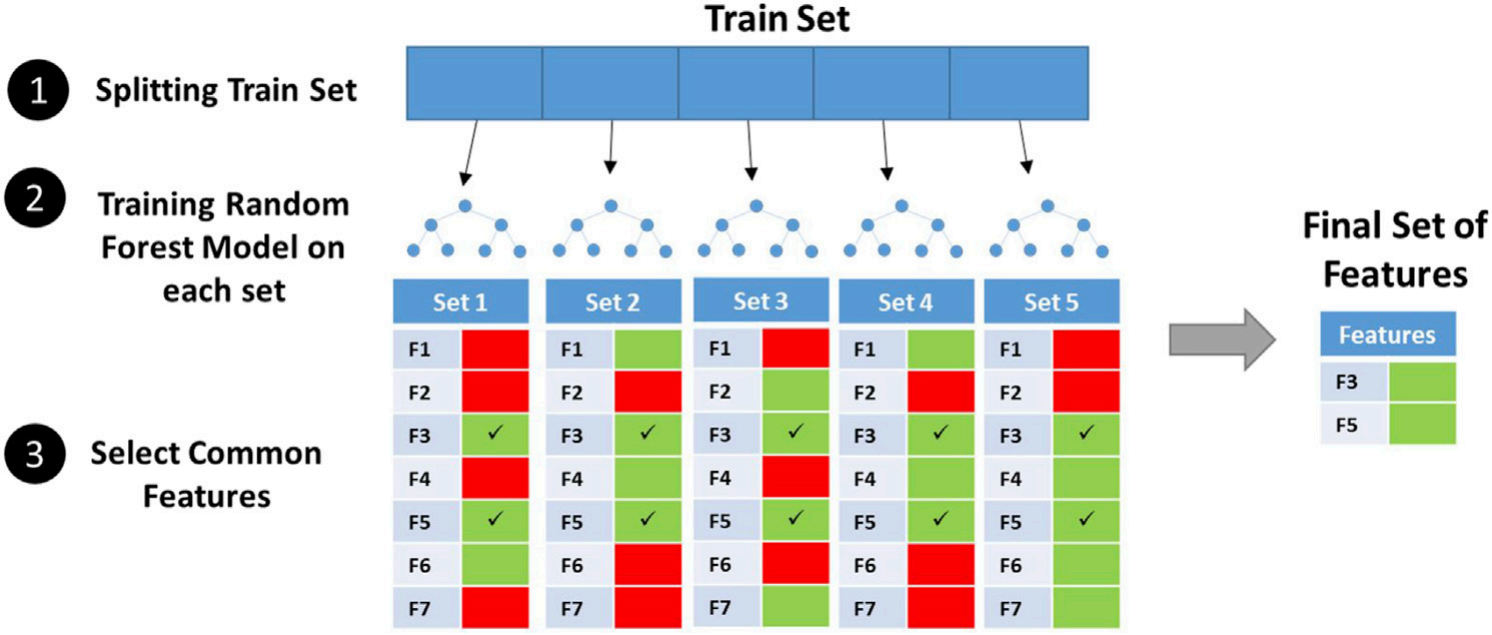
Feature selection by the cross-validation process.

Here, we used the Propy Python library to extract linguistic and physicochemical-based properties and allowed cross-validation-based feature selection to select most important properties. The performance of models on the test set before feature selection is shown in Supplementary Table S2. SVC (RBF) was the best classifier at this stage. Independent random forest models were trained on five independent sets from dataset 2's train set, and features present in all final sets were selected (Figure 2). The 1500+ properties were reduced to 51 features. The distribution of selected features among feature categories is shown in Table 3. Interestingly, all of these features were based on physicochemical properties of AMPs including net charge, molecular weight, charge density, aggregation propensity *in vivo*, distribution of charge and polarity along the peptide sequence, composition of non-polar residues, and buried residues. The relative importance of features from the random forest model is shown in Supplementary Figure S1. As can be seen in the figure, features have relatively similar importance. No linguistic-based property was found among selected features. A full list of selected features with their corresponding categories and relative importance is shown in Supplementary Table S3.

**TABLE 3:** Distribution of selected features in their categories.

Feature category	Feature sub-category	No.
Physicochemical	Net charge	1
	Molecular weight	1
	Charge density	1
	Aggregation propensity *in vivo*	1
	Physicochemical composition	4
	Physicochemical distribution	4
	Physicochemical transition	1
Autocorrelation	Geary autocorrelation	5
	Moran autocorrelation	10
	Normalized Moreau–Broto autocorrelation	6
Pseudo-amino acid composition	Pseudo-amino acid composition	4
Sequence order	Quasi-sequence order	5
	Sequence-order coupling number	8
Total		51

### 3.4 Performance of *Staphylococcus aureus*-specific activity classifier

**FIGURE 3:**
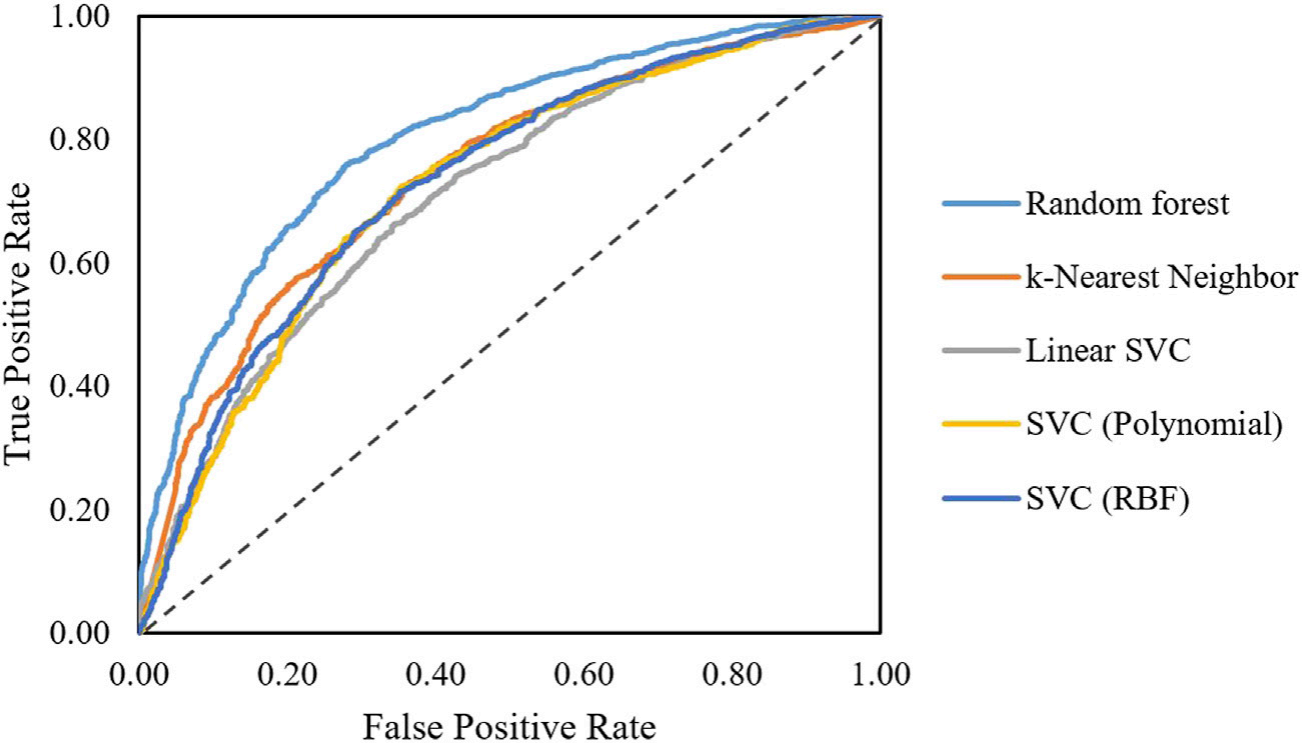
ROC curves obtained for different algorithms classifying AMPs active and non-active against *S. aureus*.

After obtaining the final feature set, classification algorithms including random forest, SVC, linear SVC, KNN, and hybrid models were trained. Performances on the training set were compared using the ROC curves (Figure 3). As shown in Figure 3, the random forest model shows a better performance compared to linear SVC and KNN models.

Table 4 shows the performance of models on the test sets. The Hamming distance between model performances shows that even models with very similar performances might get different results for the same AMP (Supplementary Figure S2). Therefore, the hybrid model made by these classifiers shows potential to perform better on the test set. Several combinations of classifiers were also used to create hybrid classifiers, and performance on test results was obtained (Supplementary Table S4). It can be seen that eclf5 (a combination of random forest, SVC polynomial, and linear SVC models) with an F1-score of 0.80 and a recall of 0.88 is the best performing classifier.

**TABLE 4:** Performance of different algorithms in classifying AMPs active against *Staphylococcus aureus*.

Algorithm	Precision	Recall	F1-score	Specificity	Accuracy	Balanced accuracy
Random forest	0.744	0.859	0.798	0.711	0.734	0.785
SVC polynomial	0.739	0.839	0.786	0.681	0.721	0.760
KNN	0.733	0.821	0.775	0.656	0.709	0.739
SVC RBF	0.741	0.777	0.759	0.624	0.699	0.700
LSVC	0.695	0.857	0.768	0.650	0.684	0.754
Hybrid	0.7415	0.8755	0.8029	0.729	0.738	0.802

### 3.5 Performance of the final shared model on the new independent set

The performance of the final constructed package of the hierarchical model was evaluated using a new dataset including AMPs and non-AMPs. Results in Table 5 show that despite low precision, the model demonstrates high sensitivity and balanced accuracy in real-world scenarios.

**TABLE 5:** Performance of the final hierarchical packaged model on an independent set.

Precision	Recall	F1-score	Specificity	Accuracy	Balanced accuracy
0.4792	1.0000	0.6479	0.7340	0.7863	0.867

## 4 Discussion

Growing AMP databases through gathering reported experimental and activity studies on AMPs has provided sufficient data for training many machine learning models for the prediction of AMP activity. Most prediction models concerning the antimicrobial activity of AMPs have been dedicated to distinguish AMPs from non-AMPs and were able to achieve high performances. However, as shown in the results of Table 1, the susceptibility to a single AMP is highly varied among different target species. Therefore, there is no guarantee that AMP candidates suggested by such predictive models will be able to show suitable activity against a specific species.

**FIGURE 4:**
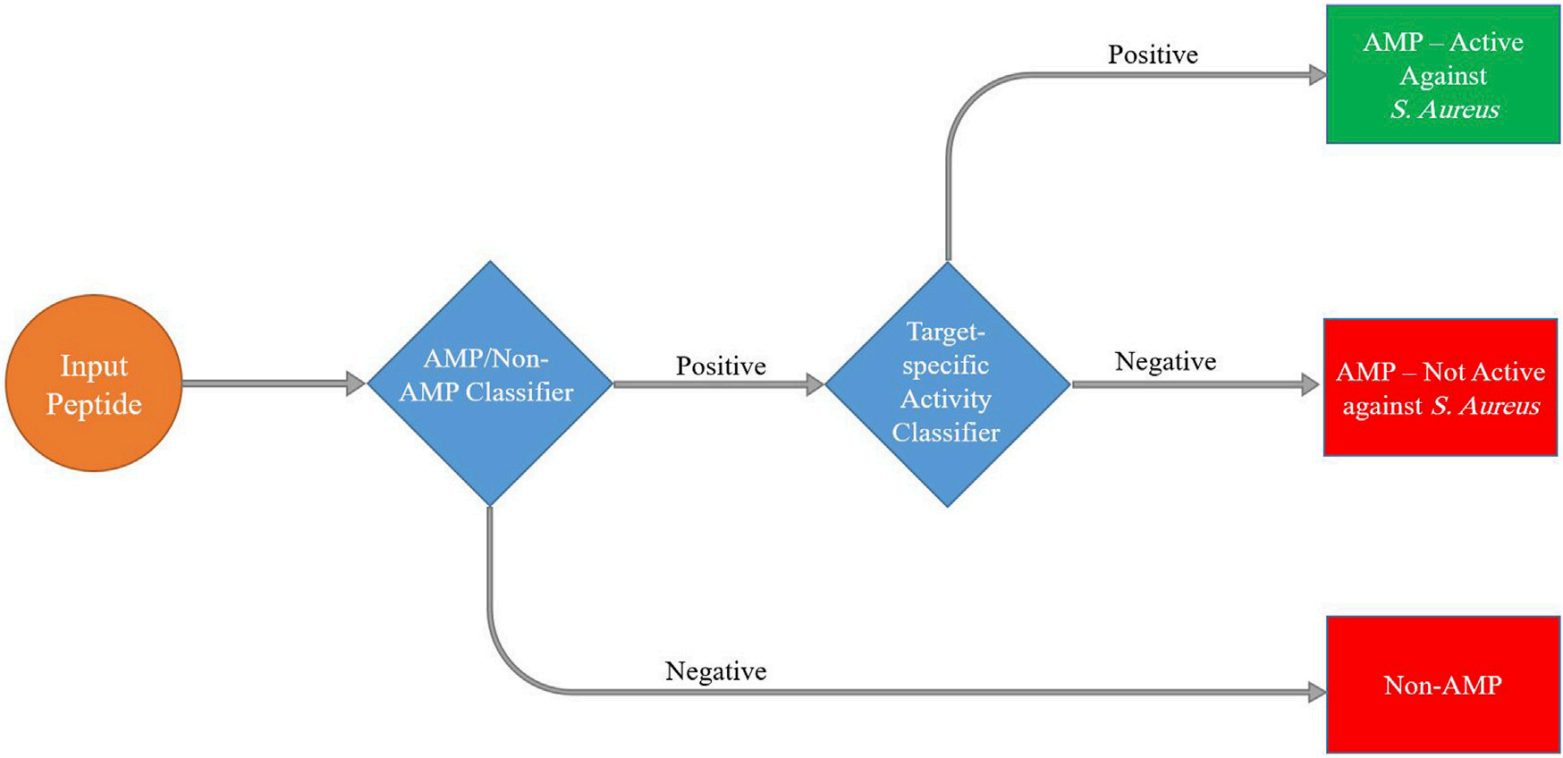
Schematic presentation of the hierarchical machine learning model pipeline to predict peptide antimicrobial activity.

In the context of our study on detecting antimicrobial peptides active against *S. aureus*, we used hierarchical models to effectively tackle the complex nature of peptide space. We trained two separate classifiers. The first level of classification involved distinguishing between two broad categories: antimicrobial peptides (AMPs) and non-antimicrobial peptides (non-AMPs). Subsequently, we utilized the second level of classification to further analyze the subset of AMPs and distinguish between those that are active against *S. aureus* (SA-AMPs) and those that are not active against *S. aureus* (non-SA-AMPs) (Figure 4).

Features extracted from peptides are based on either the amino acid letter sequence or physicochemical properties, and the performance of the final model on unseen peptides depends on the similarity of their properties with training data. However, only a very tiny portion of the sequence space is similar to the training set. Therefore, we expect classification models based on physicochemical properties of AMPs to generalize better. Notably, investigating all 51 features selected from 1,500+ features had a physicochemical nature, which is in agreement with our expectations.

The machine learning results demonstrate that the hierarchical model achieved promising performance in peptide classification. The first-level classifier showed an excellent F1-score (0.923) in differentiating between AMPs and non-AMPs, indicating its ability to capture key features that discriminate between these two categories. The second-level classifier, which focused specifically on classifying AMPs into SA-AMPs and non-SA-AMPs, also showed good performance with the F1-score (0.80) in distinguishing between these two categories.

The hierarchical approach provided several advantages over a single-classifier approach. By using a two-level classifier, we were able to first filter out non-AMPs and then further classify the remaining AMPs into two subcategories based on their activity against *S. aureus*. This hierarchical approach allows for using a more diverse library of peptides as the input compared to other studies, which have only used AMPs for training, and this is of particular interest in our research. Moreover, the hierarchical model is interpretable as it allows us to examine the performance and contributions of each level separately, making it easier to identify potential areas for improvement and fine-tuning.

Not many predictive tools are available to compare our results with theirs. Speck-Planche et al. (2016) and Vishnepolsky et al. (2022) made strain-specific predictive models to distinguish AMPs active against specific strains of bacteria, including *S. aureus*, and based on their raw performances, they are better classifiers. However, there are important considerations in practical applications and differences in the methodologies implemented. Both works only used AMPs as the training set. Therefore, their models are not familiar with non-AMP peptides. Considering this, if non-AMP peptides were to be used as input sequences, since they are not similar either to the positive or the negative set, it could result in undesirable performances. In the case of Vishnepolsky, B. et al. work, labeling AMPs was based on the MIC with the concentration unit of µg/mL, which is not biologically reasonable since the transformation to µM is necessary to allow the direct and accurate comparison of the inhibitory activities among the AMPs (Speck-Planche et al., 2016). In our work and many other recent works, µg/mL concentrations are first converted to µM (Kleandrova et al., 2016). These can limit the applicability of these models in practical applications.

It is worth noting that the performance of each level’s classifier was evaluated using independent test sets, which were distinct from the training and validation sets utilized during model development. This approach ensured that the model’s performance was generalizable and reflective of real-world scenarios. On the other hand, the final packaged hierarchical model also demonstrated strong performance on a mixed dataset of AMPs and non-AMPs, providing a significant advantage over other studies that were exclusively trained with AMPs. The results of our study suggest that the developed hierarchical model effectively classifies peptides into distinct categories, including distinguishing between SA-AMPs and non-SA-AMPs. This capability holds potential for various applications such as drug discovery, antimicrobial peptide design, and functional peptide annotation. The final package constructed here is publicly available on GitHub at: https://github.com/h-khabaz/s.aureus-AMP-activity-calculator.

## 5 Conclusion

In conclusion, we developed a hierarchical machine learning model for peptide classification, specifically targeting the classification of antimicrobial peptides against *S. aureus*. The results demonstrate the effectiveness of the hierarchical approach in accurately classifying peptides into different categories and distinguishing between AMPs active and not-active against *S. aureus*. The developed model has potential applications in various fields, including drug discovery, peptide design, and functional annotation of peptides.

## Data Availability

The datasets presented in this study can be found in online repositories. The names of the repository/repositories and accession number(s) can be found at: https://github.com/h-khabaz/s.aureus-amp-dataset.
