# Supplementary material for: Hierarchical machine learning model predicts antimicrobial peptide activity against Staphylococcus aureus
Source: Front Mol Biosci. 2023 Sep 18;10:1238509. doi: 10.3389/fmolb.2023.1238509 (PMC10544327; doi:10.3389/fmolb.2023.1238509)
Supplement: Supplementary file 1 [file DataSheet1.docx]

Supplementary Material

Hierarchical machine learning model predicts antimicrobial peptides activity against *Staphylococcus Aureus*

Hosein Khabaz, Mehdi Rahimi-Nasrabadi, Amir Homayoun Keihan^*^

*** Correspondence:** Homayoun Keihan: [ahkeihan@bmsu.ac.ir](mailto:ahkeihan@bmsu.ac.ir)

# Supplementary Figures and Tables

## Supplementary Figures


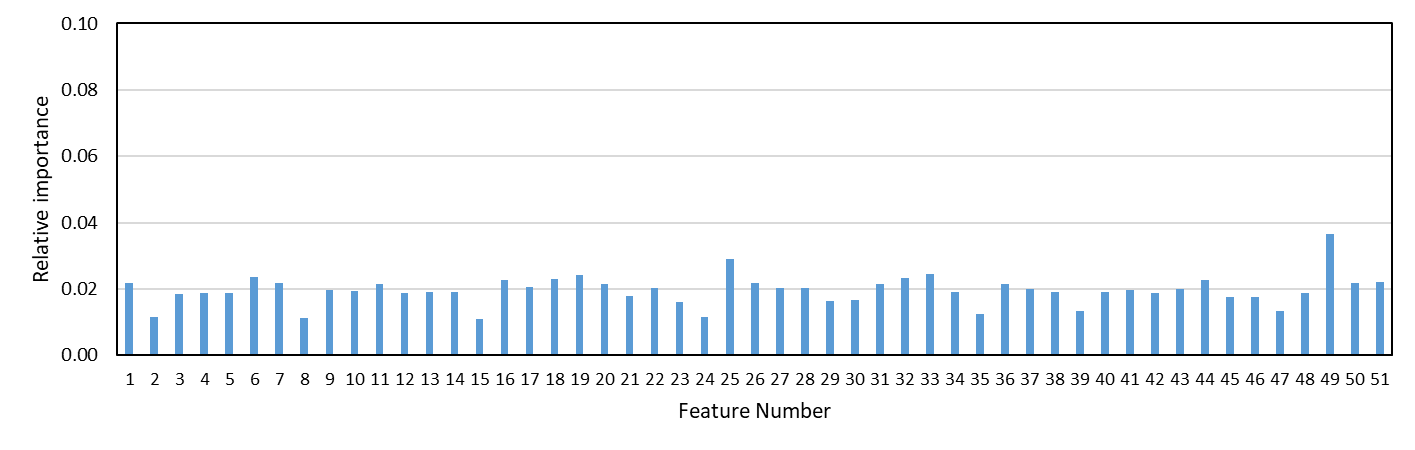


Supplementary Figure 1. Relative importance of selected features from Random forest model


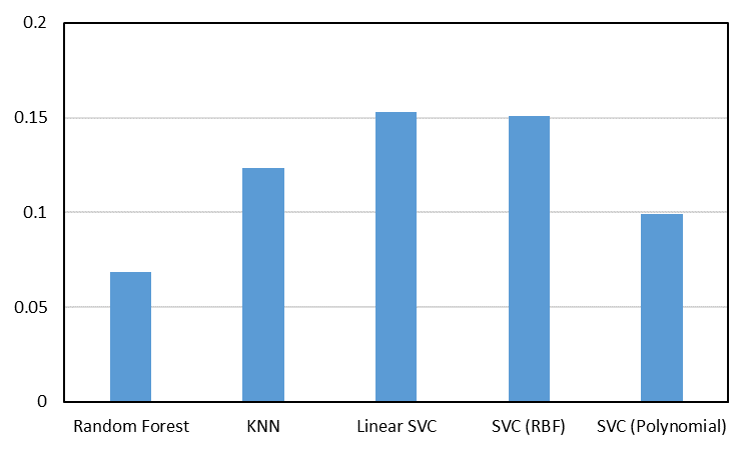


Supplementary Figure 2. The hamming distance between hybrid model and all other algorithms

## Supplementary Tables

Supplementary Table 1. Number of features calculated for each peptide in each feature categories

| Feature Category | Features | No. of Features |
| --- | --- | --- |
| Physico-chemical | Net charge | 1 |
|  | Length | 1 |
|  | Aggregation propensity *in vivo* | 1 |
|  | Charge density | 1 |
|  | Molecular weight | 1 |
|  | N-Terminal modification | 1 |
|  | C-Terminal modification | 1 |
|  | Physicochemical composition | 21 |
|  | Physicochemical transition | 21 |
|  | Physicochemical distribution | 105 |
| Amino acid composition | Amino acid composition | 20 |
|  | Dipeptide composition | 400 |
| Autocorrelation | Normalized Moreau–Broto autocorrelation | 240 |
|  | Moran autocorrelation | 240 |
|  | Geary autocorrelation | 240 |
| Sequence order | Sequence order coupling number | 90 |
|  | Quasi-sequence-order | 100 |
| Pseudo-amino acid composition | Pseudo amino acid composition I | 30 |
|  | Pseudo amino acid composition II | 20 |

Supplementary Table 2. Performance different algorithms on test set before feature selection

| Classifier | Precision | Recall | F1 Score | Accuracy | Specificity | Balanced Accuracy |
| --- | --- | --- | --- | --- | --- | --- |
| Random Forest | 0.7461 | 0.8614 | 0.7996 | 0.7368 | 0.7149 | 0.7882 |
| SVC Polynomial | 0.7363 | 0.8916 | 0.8065 | 0.7393 | 0.7477 | 0.8196 |
| KNN | 0.7354 | 0.8594 | 0.7926 | 0.7258 | 0.7021 | 0.7808 |
| SVC RBF | 0.7860 | 0.8333 | 0.8090 | 0.7601 | 0.7316 | 0.7825 |
| LSVC | 0.7769 | 0.7550 | 0.7658 | 0.7185 | 0.6336 | 0.6943 |
| Hybrid | 0.7633 | 0.8614 | 0.8094 | 0.7528 | 0.7387 | 0.8000 |

Supplementary Table 3. Importance of selected features based on Random forest classifier

| Features | importance | Category | Sub-category |
| --- | --- | --- | --- |
| molecularWeight | 0.0367 | Physico-chemical |  |
| _PolarityC1 | 0.0291 | Physicochemical | Physicochemical Composition |
| PAAC26 | 0.0244 | Pseudo-amino acid composition | Pseudo-amino acid composition |
| MoreauBrotoAuto_Mutability4 | 0.0242 | Autocorrelation | Normalized Moreau–Broto autocorrelation |
| MoranAuto_FreeEnergy7 | 0.0236 | Autocorrelation | Moran autocorrelation |
| PAAC25 | 0.0233 | Pseudo-amino acid composition | Pseudo-amino acid composition |
| MoreauBrotoAuto_Mutability1 | 0.0231 | Autocorrelation | Normalized Moreau–Broto autocorrelation |
| tausw5 | 0.0228 | Sequence order | Sequence order coupling number |
| MoreauBrotoAuto_ResidueVol1 | 0.0227 | Autocorrelation | Normalized Moreau–Broto autocorrelation |
| AggregationPropensityInVivo | 0.0221 | Physico-chemical |  |
| _PolarizabilityD3001 | 0.0219 | Physicochemical | Physicochemical Distribution |
| chargeDensity | 0.0218 | Physico-chemical |  |
| GearyAuto_Polarizability4 | 0.0218 | Autocorrelation | Geary autocorrelation |
| MoranAuto_FreeEnergy11 | 0.0217 | Autocorrelation | Moran autocorrelation |
| MoranAuto_Steric8 | 0.0216 | Autocorrelation | Moran autocorrelation |
| MoreauBrotoAuto_Mutability8 | 0.0214 | Autocorrelation | Normalized Moreau–Broto autocorrelation |
| QSOSW24 | 0.0214 | Sequence order | Quasi-sequence-order |
| PAAC22 | 0.0213 | Pseudo-amino acid composition | Pseudo-amino acid composition |
| MoreauBrotoAuto_ResidueVol5 | 0.0207 | Autocorrelation | Normalized Moreau–Broto autocorrelation |
| _SolventAccessibilityC1 | 0.0203 | Physicochemical | Physicochemical Composition |
| _SecondaryStrD2001 | 0.0203 | Physicochemical | Physicochemical Distribution |
| _ChargeD1075 | 0.0203 | Physicochemical | Physicochemical Distribution |
| QSOSW25 | 0.0200 | Sequence order | Quasi-sequence-order |
| tausw4 | 0.0199 | Sequence order | Sequence order coupling number |
| MoranAuto_ResidueVol11 | 0.0198 | Autocorrelation | Moran autocorrelation |
| tausw2 | 0.0197 | Sequence order | Sequence order coupling number |
| MoranAuto_Steric4 | 0.0194 | Autocorrelation | Moran autocorrelation |
| MoranAuto_Steric11 | 0.0192 | Autocorrelation | Moran autocorrelation |
| tausw1 | 0.0192 | Sequence order | Sequence order coupling number |
| PAAC29 | 0.0192 | Pseudo-amino acid composition | Pseudo-amino acid composition |
| MoranAuto_Mutability11 | 0.0191 | Autocorrelation | Moran autocorrelation |
| QSOSW29 | 0.0190 | Sequence order | Quasi-sequence-order |
| MoranAuto_Steric9 | 0.0189 | Autocorrelation | Moran autocorrelation |
| tausw3 | 0.0188 | Sequence order | Sequence order coupling number |
| GearyAuto_Steric6 | 0.0187 | Autocorrelation | Geary autocorrelation |
| GearyAuto_Steric9 | 0.0187 | Autocorrelation | Geary autocorrelation |
| netCharge | 0.0187 | Physico-chemical |  |
| GearyAuto_Steric4 | 0.0185 | Autocorrelation | Geary autocorrelation |
| MoreauBrotoAuto_Mutability12 | 0.0178 | Autocorrelation | Normalized Moreau–Broto autocorrelation |
| tausw9 | 0.0177 | Sequence order | Sequence order coupling number |
| tausw8 | 0.0177 | Sequence order | Sequence order coupling number |
| _PolarizabilityT13 | 0.0166 | Physicochemical | Physicochemical transition |
| _PolarityD1001 | 0.0163 | Physicochemical | Physicochemical Distribution |
| _SolventAccessibilityC3 | 0.0161 | Physicochemical | Physicochemical Composition |
| tausw13 | 0.0133 | Sequence order | Sequence order coupling number |
| QSOSW33 | 0.0133 | Sequence order | Quasi-sequence-order |
| QSOSW17 | 0.0125 | Sequence order | Quasi-sequence-order |
| GearyAuto_FreeEnergy19 | 0.0116 | Autocorrelation | Geary autocorrelation |
| _ChargeC3 | 0.0114 | Physicochemical | Physicochemical Composition |
| MoranAuto_FreeEnergy21 | 0.0114 | Autocorrelation | Moran autocorrelation |
| MoranAuto_Mutability22 | 0.0108 | Autocorrelation | Moran autocorrelation |

Supplementary Table 4. Performance hybrid classifiers with different combinations of a) Random Forest, b) SVC Polynomial, c) SVC RBF, d) LSVC, e) KNN.

| Hybrid Classifier Combinations | Precision | Recall | F1 Score | Accuracy | Specificity | Balanced Accuracy |
| --- | --- | --- | --- | --- | --- | --- |
| eclf1 (All) | 0.7367 | 0.8594 | 0.7933 | 0.7271 | 0.7034 | 0.7814 |
| eclf2 (a,b,c) | 0.7404 | 0.8534 | 0.7929 | 0.7283 | 0.6996 | 0.7765 |
| eclf3 (a,d,e) | 0.7347 | 0.8675 | 0.7956 | 0.7283 | 0.7118 | 0.7896 |
| eclf4 (a,b,e) | 0.7431 | 0.8594 | 0.797 | 0.7332 | 0.7095 | 0.7845 |
| eclf5 (a,b,d) | 0.7415 | 0.8755 | 0.8029 | 0.7381 | 0.7293 | 0.8024 |
| eclf6 (a,c,d) | 0.7363 | 0.8635 | 0.7948 | 0.7283 | 0.7082 | 0.7858 |
| eclf7 (a,c,e) | 0.7459 | 0.8313 | 0.7863 | 0.7246 | 0.6794 | 0.7553 |
| eclf8 (b,c,d) | 0.7353 | 0.8534 | 0.79 | 0.7234 | 0.6946 | 0.7740 |
| eclf9 (b,c,e) | 0.7478 | 0.8454 | 0.7936 | 0.7319 | 0.6969 | 0.7711 |
| eclf10 (b,d,e) | 0.734 | 0.8755 | 0.7985 | 0.7307 | 0.7220 | 0.7987 |
| eclf11 (c,e,d) | 0.732 | 0.8394 | 0.782 | 0.7148 | 0.6748 | 0.7571 |

Supplementary Table 5. Hyper-parameters obtained for each classifier using grid search

| Level | Classifier | Hyper-parameter | Optimized Value |
| --- | --- | --- | --- |
| AMP/non-AMP classifier | Random Forest | No. of estimators | 400 |
|  |  | Max Features | 2 |
|  |  | Max Depth | 10 |
|  | SVC RBF | c | 500 |
|  |  | gamma | 0.95 |
|  | KNN | No. of neighbors | 5 |
|  |  | Weights | Uniform |
|  | Naïve Bayas | var smoothing | 1.00E-18 |
| *S. Aureus* specific activity classifier | Random Forest | No. of estimators | 400 |
|  |  | Max Features | 12 |
|  |  | Max Depth | 15 |
|  | SVC Polynomial | c | 1 |
|  |  | gamma | 0.7 |
|  | KNN | No. of neighbors | 7 |
|  |  | Weights | distance |
|  | SVC RBF | c | 200 |
|  |  | gamma | 0.9 |
|  | LSVC | c | 1 |
